# Acquisition of a single EZH2 D1 domain mutation confers acquired resistance to EZH2-targeted inhibitors

**DOI:** 10.18632/oncotarget.5066

**Published:** 2015-09-02

**Authors:** Theresa Baker, Sujata Nerle, Justin Pritchard, Boyang Zhao, Victor M. Rivera, Andrew Garner, Francois Gonzalvez

**Affiliations:** ^1^ ARIAD Pharmaceuticals, Inc. Cambridge, MA 02139, USA

**Keywords:** EZH2, drug resistance, mutation, epigenetics, cancer

## Abstract

Although targeted therapies have revolutionized cancer treatment, overcoming acquired resistance remains a major clinical challenge. EZH2 inhibitors (EZH2i), EPZ-6438 and GSK126, are currently in the early stages of clinical evaluation and the first encouraging signs of efficacy have recently emerged in the clinic. To anticipate mechanisms of resistance to EZH2i, we used a forward genetic platform combining a mutagenesis screen with next generation sequencing technology and identified a hotspot of secondary mutations in the EZH2 D1 domain (Y111 and I109). Y111D mutation within the WT or A677G EZH2 allele conferred robust resistance to both EPZ-6438 and GSK126, but it only drove a partial resistance within the Y641F allele. EZH2 mutants required histone methyltransferase (HMT) catalytic activity and the polycomb repressive complex 2 (PRC2) components, SUZ12 and EED, to drive drug resistance. Furthermore, D1 domain mutations not only blocked the ability of EZH2i to bind to WT and A677G mutant, but also abrogated drug binding to the Y641F mutant. These data provide the first cellular validation of the mechanistic model underpinning the oncogenic function of WT and mutant EZH2. Importantly, our findings suggest that acquired-resistance to EZH2i may arise in WT and mutant EZH2 patients through a single mutation that remains targetable by second generation EZH2i.

## INTRODUCTION

Polycomb repressive complex 2 (PRC2) is a multi-subunit complex that selectively silences transcription of target genes, through the specific methylation of lysine 27 of histone H3 (H3K27). EZH2, catalytic subunit of PRC2, utilizes its SET domain to transfer a methyl group from S-adenosyl methionine (SAM) to H3K27. Several genetic contexts confer dependency on EZH2 activity in cancer and lead to accumulation of tri-methylated H3K27 (H3K27me3). Both amplification and overexpression of WT EZH2 have been implicated in tumorigenesis, and associated with progressive disease stages and prognoses in several cancers [1–3]. More recently, WT EZH2 has emerged as a vulnerability in cancer associated with inactivation in the chromatin remodeling SWI/SNF complex, such as SMARCB1/INI1 mutated rhabdoid cancer and ARID1A mutated ovarian cancers [4, 5]. Three hotspots of activating SET domain mutations drive H3K27me3 and confer oncogenic addiction in non-Hodgkin lymphoma; Y641, the major incident hotpot, is present in 14–22% of germinal center diffuse large B-cell lymphoma (GCB-DLBCL) and 7–22% of follicular lymphoma (FL), while A677G and A687G are lowest prevalent hotspots, and present in around 1–3% in GCB-DLBCL and FL [6–9]. These heterozygous mutations alter the substrate specificity of PRC2, and favor the generation of H3K27me3. Biochemical data suggest that Y641 mutant requires the WT allele to generate H3K27me3, while A677G functions independently of its WT counterpart [7, 10]. The identification of these gain of function mutations provided validation for the oncogenic potential of EZH2 and led to a surge of interest in exploring the therapeutic potential of EZH2 as a cancer target. As a result, potent and selective EZH2-targeted small molecule inhibitors (EZH2i) have recently been generated [11–13]; all compete with SAM binding to the SET domain and thus inhibit PRC2 histone methyl transferase (HMT) activity [14, 15]. In preclinical models, EZH2i decrease global H3K27me3 levels and induce regression of WT and mutant EZH2 tumors [5, 13, 16]. The most advanced compounds, EPZ-6438 (E7438) and GSK126 (GSK2816126), are currently being tested in lymphoma patients harboring WT or EZH2 mutations (NCT01897571 and NCT02082977). In a phase 1 clinical trial, EPZ-6438 showed durable objective responses as monotherapy in both DLBCL and rhabdoid INI1-deficient patients [17].

Missense mutations that adversely impact drug binding are commonly responsible for acquired resistance to targeted therapies across a broad range of target classes; from oncogenic kinases targeted by small molecule inhibitors (e.g. imatinib and T315I in BCR-ABL), to growth factor receptors targeted by antibodies (e.g. cetuximab and S429R in EGFR) [18–20]. The identification and characterization of drug-induced resistance mutations, either from relapsed patients, or resistant cell lines, has guided the design of second generation drugs that are unaffected by resistant mutants and consequently induce profound clinical responses in relapsed patients [21–23]. Since EZH2i have recently begun to exhibit encouraging clinical efficacy in WT EZH2 patients [17], it is crucial to anticipate the resistance mechanisms to EZH2 targeted therapy. Here, we used forward genetics to identify a mechanism of acquired resistance to EZH2i.

## RESULTS

**Figure 1:**
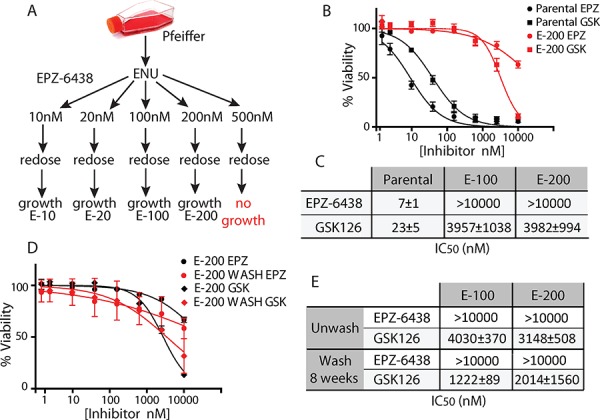
Generation of cells resistant to EZH2i **A.** Experimental strategy for generating EPZ-6438 resistant Pfeiffer cells. **B.** and **C.** Sensitivity of E-200 Pfeiffer cells to a dose response of EPZ-6438 (EPZ) and GSK126 (GSK). **D.** and **E.** E-200 cells grown after withdrawal of EPZ for 8 weeks (WASH) were treated with a dose response of EZH2i and assayed for cell viability. All IC_50_ values (±S.D.) are calculated from three independent experiments.

To identify mechanisms of acquired EZH2i resistance, we used the B-cell lymphoma Pfeiffer cell line as a model system, since it naturally harbors an EZH2 A677G mutation that renders it highly sensitive to EZH2i [7, 11, 12]. Pfeiffer cells were mutagenized and subsequently incubated with varying concentrations of EPZ-6438. After 4 weeks of incubation, drug-resistant outgrowth was observed at EPZ-6438 concentrations as high as 200 nM (Figure 1A). Cells derived with either 100 or 200 nM EPZ-6438 (termed E-100 and E-200, respectively) were highly resistant to both EPZ-6438 and GSK126, with cell viability IC_50_ values being increased by at least 1000-fold (Figures 1B, 1C, and S1A–S1C). Importantly, EZH2i resistance was maintained after 2 months of drug withdrawal (Figures 1D, 1E, and S1C), suggesting a genetic mechanism of resistance.

**Figure 2:**
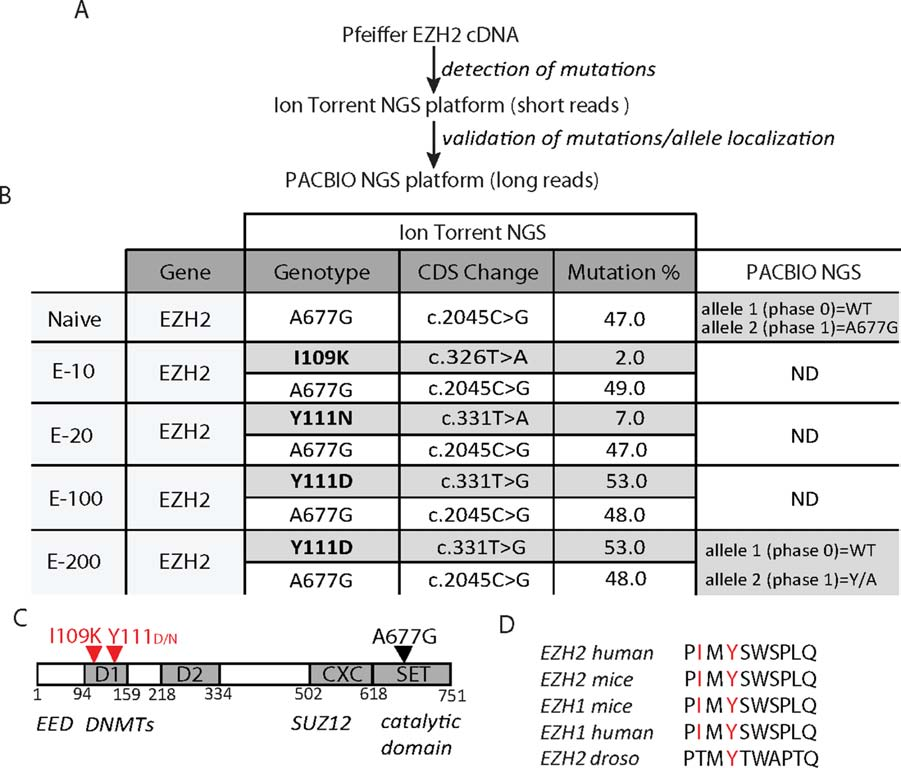
Cells resistant to EZH2i acquire mutations in the EZH2 D1 domain **A.** Schematic illustration of the next generation sequencing (NGS) approach to identify EZH2 mutants. **B.** Summary of NGS sequencing of EZH2 in Pfeiffer-resistant cells with Ion Torrent and Pacbio platforms. **C.** EZH2 domain structure with location of primary (black) and resistant mutations (red). D1, domain 1; D2, domain 2; CXC, cysteine rich domain; SET, methyl transferase catalytic domain; EED, embryonic ectoderm development interaction region; DNMT, DNA methyl transferase interaction region; SUZ12, suppressor of zeste 12 interaction region. **D.** Species alignment of human I109 and Y111 EZH2.

We next screened for the presence of drug-resistant EZH2 mutations using two complementary next generation sequencing (NGS) techniques (Figure 2A): ion semiconductor sequencing (Ion Torrent) and single molecule real-time sequencing (PacBio). As expected, both parental and drug-resistant Pfeiffer cells contained a heterozygous EZH2 A677G mutation. Surprisingly, no additional SET domain mutations were identified in the resistant cells (Figure 2B). However, in both E-100 and E-200, a high frequency EZH2 Y111D missense mutation (Figure 2B) was identified. Furthermore, sequencing of the two resistant cell lines derived with lower EPZ-6438 concentrations (E-10 and E-20) identified two further, low frequency missense mutations: I109K and Y111N (Figure 2B). I109 and Y111 are both highly conserved residues within the N-terminal D1 domain of EZH2 (Figure 2C and 2D). Interestingly, EZH2 Y111D was present at a similar Ion Torrent sequencing read frequency to the primary A677G mutation, suggesting that both mutations co-exist within the same *EZH2* allele. This hypothesis was directly confirmed by single molecule real-time sequencing, which demonstrated that E-200 cells harbored Y111D and A677G in the same allele (Figure 2B and S2). Collectively, these data suggest that the EZH2 D1 domain, and particularly the conserved residues I109 and Y111, is a hotspot for mutations that can confer resistance to EZH2-targeted therapies.

**Figure 3:**
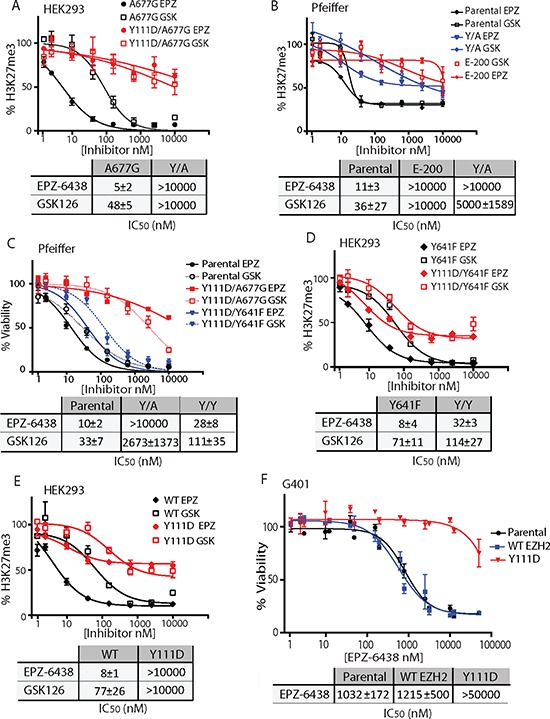
A single EZH2 D1 domain mutation confers resistance to EZH2i **A, D,** and **E.** Evaluation of H3K27me3 in EZH2 mutants stably-expressing HEK293 following treatment with EPZ-6438 (EPZ) and GSK 126 (GSK). **B.** Pfeiffer cells stably-expressing Y111D/A677G EZH2 mutant were treated with a dose response of EPZ-6438 and GSK126 and assayed for H3K27me3. **C.** Pfeiffer cells stably-expressing Y111D/A677G and Y111D/Y641F EZH2 mutants were treated with a dose response of EPZ-6438 and GSK126 and assayed for viability. **F.** G401 cells stably-expressing WT and Y111D EZH2 mutant were treated with a dose response of EPZ-6438 and assayed for viability. (A–F) IC_50_ values (±S.D.) were calculated from three independent viability assays or H3K27me3 alpha-LISA experiments.

We examined the functional impact of D1 domain mutations by stably expressing various EZH2 mutants in HEK293 cells. Ectopic expression of EZH2 mutants did not affect the level of other PRC2 components, SUZ12 and EED (Figure S3A). As previously reported [7], A677G EZH2, substantially reduced H3K27 di-methylation (H3K27me2) and increased H3K27 tri-methylation (H3K27me3) (Figure S3A). Of note, WT EZH2 induced a slight increase in H3K27me3 but did not affect H3K27me2. Importantly, A677G-driven H3K27me3 was highly sensitive to EZH2 inhibition (Figure 3A and S3B), thus validating HEK293 cells as a suitable model to study EZH2 HMT function. Expression of Y111D/A677G EZH2 induced similar H3K27me3, but in contrast to A677G alone, H3K27me3 activity was completely insensitive to EPZ-6438 and GSK126 inhibition (Figures 3A and S3B). I109K/A677G EZH2 mutants were also resistant to EZH2i (Figures S3B and S3C). However, the degree of drug resistance imparted by I109K was significantly lower than that conferred by Y111D, thus rationalizing why this mutation was only observed at low dose of EPZ-6438 (Figure 2B). Cumulatively, these data demonstrate that D1 domain mutations block the ability of EZH2i to inhibit HMT activity.

To directly assess the ability of D1 domain mutations to confer drug-resistance, we stably expressed EZH2 mutants in Pfeiffer cells and assessed their sensitivity to EZH2i. Similar to HEK293, Y111D mutation did not affect EED, SUZ12 and global H3K27me3 levels in E-200 and Y/A expressing Pfeiffer cells (Figure S3D). Expression of WT or Y111D in Pfeiffer cells did not affect sensitivity to EZH2i (Figure S4B). Strikingly, Y111D/A677G compound mutations conferred both growth and H3K27 tri-methylation resistance to EPZ-6438 and GSK126 (Figure 3B and 3C), at a level similar to E-100 and E-200 cells. Notably, the D1 domain mutation Y111D also conferred drug resistance in the context of the Y641 oncogenic mutation hotspot. However, in contrast to the degree of resistance imparted by Y111D/A677G mutant (1000 fold shift in IC_50_ values), Y111D/Y641F induced a 3–4 fold increase in IC_50_ values in both growth and H3K27 tri-methylation (Figure 3C, 3D, and S3E). Then, we examined the functional impact of the D1 domain mutation on WT EZH2-driven cells. HEK293 cells stably expressing WT EZH2 were highly sensitive to EZH2i (Figure 3E and S3F). Y111D mutation blocked the ability of EZH2i to inhibit WT EZH2 activity to a similar extent than in A677G. To directly assess the ability of the Y111D mutation to drive resistance in the context of WT EZH2, we stably expressed the Y111D mutant in a WT EZH2-driven G401 cell line. The malignant rhaddoid tumor G401 cells harbor homozygous inactivating mutations in SMARCB1/INI1 that render them depend on WT EZH2 activity for survival [5]. In keeping with HEK293 data, expression of Y111D in G401 conferred complete resistance to EPZ-6438 (Figure 3F). Thus, acquisition of a single mutation within D1 domain (Y111D) is sufficient to drive robust resistance to EZH2i in the context of the WT and A677G mutant EZH2, but only confers partial resistance in the context of the Y641 hotspot.

**Figure 4:**
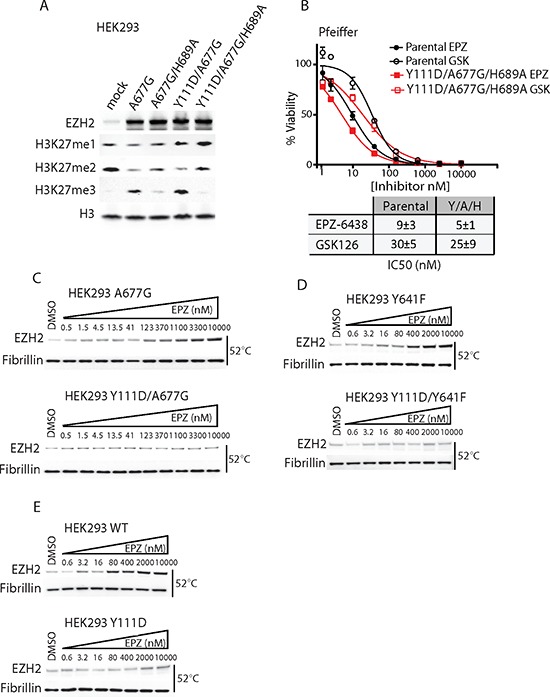
EZH2 D1 domain mutants require an active SET domain to drive resistance and inhibit drug binding both in WT and mutant EZH2 **A.** HEK293 cells were transiently transfected with A677G (A), A677G/H689A (A/H), Y111D/A677G (Y/A) or Y111D/A677G/H689A (Y/A/H) EZH2 mutants and analyzed by immunoblot for H3K27 methylation status. **B.** Pfeiffer cells stably expressing Y111D/A677G/H689A were treated with a dose response of EPZ-6438 and GSK126 and assayed for cell viability. Lower insert displays IC_50_ values (±S.D.) calculated from three independent experiments. **C–E.** Measure of target engagement by CETSA. HEK293 cells stably expressing EZH2 WT or mutants were incubated with DMSO or incremental dose of EPZ-6438 for 3 hr and heated at 52°C. Nucleoplasmic lysates were analyzed by immunoblot for EZH2 level.

Next, we asked whether EZH2i resistance was dependent on EZH2 enzymatic activity by introducing the catalytically deficient H689A SET domain mutation into A677G and Y111D/A677G mutants [24]. As previously reported, H689A significantly reduced the ability of EZH2 A677G to generate H3K27me3 (Figure 4A). Similarly, H689A also abrogated the catalytic activity of EZH2 Y111D/A677G, indicating that Y111D/A677G directly tri-methylates H3K27 through its SET domain. Moreover, the catalytically inactive EZH2 mutant Y111D/A677G/H689A was unable to confer EZH2i resistance to Pfeiffer cells (Figure 4B). Thus, Y111D/A677G requires a catalytically competent SET domain to drive proliferation in the presence of EZH2i. Of note, Y111D alone had no impact on EZH2 HMT activity, indicating that it did not confer resistance through a gain-of-activity mechanism (Figure S3A). Taken together, these results demonstrate that EZH2 D1 domain mutants require HMT activity to drive drug-resistance, rather than recruiting another HMT (e.g. EZH1) to the PRC2 complex.

Since the D1 domain mutation did not affect the level of the core PRC2 complex components (Figure S3A, S3B, and S3D), we examined whether Y111D/A677G was reliant upon EED and SUZ12 to effectively function as an HMT. Similar to EZH2 A677G, Y111D/A677G was dependent on EED and SUZ12 subunits to perform H3K27me3 (Figure S4A), demonstrating that there is no overall change in subunit dependency. There is evidence that EZH2 recruits DNA methyltransferases (DNMTs) to the PRC2 complex via its D1 domain and regulates promoter methylation of PRC2 target genes. However, it is not currently thought that DNMTs directly influence EZH2 HMT activity [25]. In keeping with this, silencing DNMT1, 3A, or 3B had no overall impact on the HMT activity of Y111D/A677G EZH2 (Figure S4A). These data imply that the D1 domain mutations do not significantly affect the assembly of the minimal PRC2 components (EZH2, EED, and SUZ12) required to undertake H3K27 methylation.

Finally, we performed a cellular thermal shift assay (CTSA) to directly measure the impact of the D1 domain mutation on drug binding. Consistent with a previous report [26], 52°C was determined as the optimal temperature at which EZH2 protein is stabilized by EZH2i (data not shown). EPZ-6438 induced a dose-dependent thermal-stabilization of WT, A677G, and Y641F mutant EZH2 in HEK293 cells, thus confirming the ability of the drug to directly bind to WT and both mutant EZH2 in cells (Figure 4C, 4D, and 4E). Importantly, Y111D totally inhibited drug-induced stabilization of WT, A677G, and Y641F mutant EZH2 (Figure 4C, 4D, and 4E). Thus, acquisition of Y111D is sufficient to completely block the binding of EZH2i to WT and mutant EZH2 (A677G or Y641F) in a cellular context.

## DISCUSSION

Using forward genetics, we have discovered a novel hotspot of EZH2 missense mutations (Y111 and I109) that conferred resistance to EZH2i. Surprisingly, this mutation hotspot lies outside the EZH2 catalytic region in its D1 domain, suggesting a unique allosteric mechanism of drug resistance. In a cellular context, acquisition of a single D1 domain mutation within the WT or A677G EZH2 alleles was sufficient to drive robust resistance, but it only conferred partial resistance in the context of the Y641 allele. However, Y111D completely abrogated the binding of EZH2i to WT and both EZH2 mutants (A677G and Y641F). That Y111D/Y641F only induced moderate resistance despite fully blocking drug binding supports the mechanistic model of coupling between both WT EZH2 and Y641, which was previously anticipated from biochemical studies [7, 10]. *In vitro*, WT EZH2 displayed high affinity toward H3K27 and H3K27me1, but much lower affinity toward H3K27me2. Therefore, WT EZH2 is predicted to generate low cellular levels of H3K27me3 at steady state and allow for the accumulation of H3K27me3 when overexpressed. A677G is predicted to function independently of its WT counterpart and to efficiently catalyze the three methylation steps to generate H3K27me3 in cells. Importantly, our data showed that a single D1 domain mutation is sufficient to confer complete resistance in WT and A677G-driven cells, thus confirming that both WT and A677G mutant EZH2 can independently support all the three steps leading to H3K27me3. In contrast, biochemical studies suggested that Y641F requires WT EZH2 to perform the first methylation reaction (H3K27 to H3K27me1). Our cellular data in Y641F-driven cells are supportive of this *in vitro*-based model. Since WT EZH2 is sensitive to EZH2i, Y111D/Y641F can only drive moderate cellular drug-resistance, despite being refractory to drug binding. Therefore, we provide the first cellular validation for the mechanistic model underpinning the oncogenic function of WT, Y641F, and A677G mutant EZH2 [7, 10]. Gibaja *et al.* have recently identified a double mutation, Y111L in the WT allele and Y661D in the Y641 allele, in a Y641N-driven KARPAS cell line that acquired robust resistance to EZH2i [27]. In biochemical assays, both mutants retained catalytic activity and fully abrogated drug binding. Based on these data, they proposed that mutations in both WT and Y641 alleles are required to drive complete EZH2i resistance in a Y641N-driven cell line. Surprisingly, a single Y111L mutation was also found associated to a robust resistance phenotype in KARPAS cells (IC50 > 10 μM). Taken together, these findings suggest that acquisition of a single D1 domain mutation is sufficient to drive resistance to EZH2i in WT, Y641, and A677-driven cells.

ENU mutagenesis screens have successfully anticipated resistant mutations that emerged in patients treated with targeted therapies: T315I BCR-ABL mutant for imatinib [28], T790M EGFR mutant for gefitinib [29], L1196M ALK mutant for crizotinib [30]. Given the predictive power of such approach, these data have several important clinical implications. Our results predict the acquisition of a single D1 domain mutation in both WT and mutant EZH2 patients treated with EZH2i, which is more likely to emerge in the clinic than a double mutation. This is potentially clinically important considering that the first signs of efficacy with EPZ-6438 have been reported in patients harboring WT EZH2 [17]. We therefore recommend not to limit to the conventional screening for catalytic domain mutants, but to screen EZH2i relapsed patients for EZH2 D1 domain mutations. Furthermore, the identification of this mutational mechanism of resistance confirms that EZH2 can function as an oncogenic driver in GCB-DLBCL, thus further validating the therapeutic potential of EZH2-targeted agents.

Importantly, Y111D remained reliant upon an active EZH2 SET domain and intact PRC2 complex to drive resistance, indicating that targeting either the SET domain via small molecules or disrupting the EED/EZH2 interaction via peptides [31] are viable drug discovery strategies for second generation EZH2 inhibitors. Although the structure of the EZH2 SET domain has recently been solved [14, 15], little structural or biochemical information is currently available to rationalize the discovered functional interplay between the D1 and SET domains. Ultimately, it will be important to understand how the various EZH2 domains regulate its activity and/or drug sensitivity, in order to determine the most effective strategy to therapeutically target EZH2, while minimizing the emergence of acquired resistance.

## MATERIALS AND METHODS

### Cell lines and reagents

Pfeiffer were obtained from ATCC. HEK293 cells were obtained from Thermo Scientific. Pfeiffer cells, were cultured in RPMI 1460 (Life Technologies, 22400105). HEK293 cells were cultured in Dulbecco's modified Eagle's medium (DMEM) (Life Technologies, 11965118). Culture media were supplemented with 10% of FBS (Life Technologies, 26140129).

Antibodies for immuno-blotting: EZH2 (CST, #5246), Histone H3 (CST, #4499), Histone H3 Lysine 27 Methyl 2(CST, #9728), Histone H3 Lysine 27 Methyl 3(CST, #9733). Antibody Histone H3 Lysine 27 Methyl 1 (EMD Millipore, #07–448). Anti-rabbit secondary antibody stored in 50% glycerol (Jackson Immunoresearch Laboratories, Inc., 205–035-108). GSK 126 and EPZ-6438 were purchased from AdooQ and Xcess Biosciences, respectively. Compounds were dissolved to 10 mM in dimethyl sulfoxide (DMSO).

### Expression construct

Cloning of EZH2 CDS into the pLVX.Puro vector (Clontech) was performed by Genscript. Mutations of EZH2 cDNA were generated using the Strategene Quickchange Site-Directed Mutagenesis kit (La Jolla, CA). Stable expression of EZH2 mutants in Pfeiffer or HEK293 cells was performed by lentiviral transduction according to manufacturer's instructions (Thermo Scientific).

For transient expression experiments, HEK293 cells were transfected using Lipofectamine 2000 (Invitrogen) according to the manufacturer's protocol.

### siRNA transfection

The siRNA oligonucleotide pools were from Dharmacon (GE Healthcare)

On-TARGET plus SMARTpool siRNA EZH2 catalog #L-004218–00-0005

On-TARGET plus SMARTpool siRNA EED catalog #L-017581–00-0005

On-TARGET plus SMARTpool siRNA SUZ12 catalog #L-006957–00-0005

On-TARGET plus SMARTpool siRNA DNMT1 catalog #L-004605–00-0005

On-TARGET plus SMARTpool siRNA DNMT3A catalog #L-006672–00-0005

On-TARGET plus SMARTpool siRNA DNMT3B catalog #L-006395–00-0005

HEK293 cells were transfected with siRNA using RNAiMAX according to manufacturer's protocol.

### Viability assay

Optimal cell seeding density was determined by performing the growth curves of the cell lines over 6 days. Cells were then plated and dosed with an 8-point, 4-fold dilution series starting at 10 μM of GSK 126 and EPZ-6438 using the Tecan HP D300 according to manufacturer's directions. 0.1% DMSO was used as a vehicle control. Cells were incubated for 6 days at 37°C and viability was measured using Cell-Titer-Glo (Promega) according to manufacturer's specifications. Dose response curves were determined and used to calculate IC_50_ values using XLFIT.

### Immuno-blot analysis of H3K27 levels

Cells were dosed with GSK 126 or EPZ-6438 at 2 μM, 0.5 μM, 0.2 μM, 0.05 μM, 0.02 μM, 0.005 μM, 0.002 μM, or DMSO 1:1000. After 72 hours, cells were lysed in 2 mL of RIPA buffer and chromatin solubilized by sonication (Thermo Scientific). Lysates were then assayed for protein content with BCA kit (Thermo Scientific) and analyzed by immuno-blot.

### Measurement of cellular levels of H3K27me3

Cells were plated at 500 cells/well in 10% DMEM, 50 uL/well in 384 well plates. Cells were dosed with Tecan D300 started at 10 μM, 4-fold dilution, 8 point curve series of GSK 126 and EPZ-6438. DMSO (0.1%) was used as a vehicle control. Plates were incubated for 72 hours at 37°C. Cellular levels of H3K27me3 were measured using AlphaLISA tri-methyl histone H3K27 cellular detection kit (Perkin Elmer) according to manufacturer's instructions. Plates were read using AlphaLISA protocol on Envision (Perkin Elmer). Dose response curves were determined and used to calculate IC_50_ values using XLfit.

### *In vitro* mutagenesis screen

Pfeiffer cells were treated overnight with 100 μg/mL of N-ethyl-N-nitrosourea (ENU; Sigma Aldrich, St. Louis, MO, USA) and then distributed into flasks containing 10, 20, 100, 200 and 500 nM of EPZ-6438. After 7 days, cells were dosed again with fresh compound and grown until outgrowth (3 to 4 weeks). EZH2 cDNA from outgrown cells was amplified by RT-PCR using Transcriptor High Fidelity cDNA Synthesis Kit (Roche, 05081955001) and submitted for NGS. Ion semiconductor sequencing (Ion Torrent) and single molecule real time sequencing (PacBio) were performed at Molecular MD and Expression analysis (Quintiles), respectively.

### Cellular Thermal Shift Assay (CTSA)

CTSA was carried out as previously described [26]. HEK293 stably-expressing EZH2 constructs were seeded overnight and treated with incremental doses of EZH2 inhibitor for 3 hrs. The cells were harvested in PBS, heated at 52°C for 3 minutes and then cooled down at room temperature for 3 minutes. Nucleoplasmic lysates were extracted following the method of Bradley et al and analyzed by SDS-PAGE followed by immuno-blot with relevant antibodies.

## SUPPLEMENTARY FIGURES



## References

[1] Kleer CG, Cao Q, Varambally S, Shen R, Ota I, Tomlins SA, Ghosh D, Sewalt RG, Otte AP, Hayes DF, Sabel MS, Livant D, Weiss SJ (2003). EZH2 is a marker of aggressive breast cancer and promotes neoplastic transformation of breast epithelial cells. Proc Natl Acad Sci U S A.

[2] Varambally S, Dhanasekaran SM, Zhou M, Barrette TR, Kumar-Sinha C, Sanda MG, Ghosh D, Pienta KJ, Sewalt RG, Otte AP, Rubin MA, Chinnaiyan AM (2002). The polycomb group protein EZH2 is involved in progression of prostate cancer. Nature.

[3] Wagener N, Macher-Goeppinger S, Pritsch M, Husing J, Hoppe-Seyler K, Schirmacher P, Pfitzenmaier J, Haferkamp A, Hoppe-Seyler F, Hohenfellner M (2010). Enhancer of zeste homolog 2 (EZH2) expression is an independent prognostic factor in renal cell carcinoma. BMC Cancer.

[4] Bitler BG, Aird KM, Garipov A, Li H, Amatangelo M, Kossenkov AV, Schultz DC, Liu Q, Shih Ie M, Conejo-Garcia JR, Speicher DW, Zhang R (2015). Synthetic lethality by targeting EZH2 methyltransferase activity in ARID1A-mutated cancers. Nat Med.

[5] Knutson SK, Warholic NM, Wigle TJ, Klaus CR, Allain CJ, Raimondi A, Porter Scott M, Chesworth R, Moyer MP, Copeland RA, Richon VM, Pollock RM, Kuntz KW (2013). Durable tumor regression in genetically altered malignant rhabdoid tumors by inhibition of methyltransferase EZH2. Proc Natl Acad Sci U S A.

[6] Bodor C, Grossmann V, Popov N, Okosun J, O'Riain C, Tan K, Marzec J, Araf S, Wang J, Lee AM, Clear A, Montoto S, Matthews J (2013). EZH2 mutations are frequent and represent an early event in follicular lymphoma. Blood.

[7] McCabe MT, Graves AP, Ganji G, Diaz E, Halsey WS, Jiang Y, Smitheman KN, Ott HM, Pappalardi MB, Allen KE, Chen SB, Della Pietra A, Dul E (2012). Mutation of A677 in histone methyltransferase EZH2 in human B-cell lymphoma promotes hypertrimethylation of histone H3 on lysine 27 (H3K27). Proc Natl Acad Sci U S A.

[8] Morin RD, Johnson NA, Severson TM, Mungall AJ, An J, Goya R, Paul JE, Boyle M, Woolcock BW, Kuchenbauer F, Yap D, Humphries RK, Griffith OL (2010). Somatic mutations altering EZH2 (Tyr641) in follicular and diffuse large B-cell lymphomas of germinal-center origin. Nat Genet.

[9] Ryan RJ, Nitta M, Borger D, Zukerberg LR, Ferry JA, Harris NL, Iafrate AJ, Bernstein BE, Sohani AR, Le LP (2011). EZH2 codon 641 mutations are common in BCL2-rearranged germinal center B cell lymphomas. PLoS One.

[10] Swalm BM, Knutson SK, Warholic NM, Jin L, Kuntz KW, Keilhack H, Smith JJ, Pollock RM, Moyer MP, Scott MP, Copeland RA, Wigle TJ (2014). Reaction Coupling between Wild-Type and Disease-Associated Mutant EZH2. ACS Chem Biol.

[11] Garapaty-Rao S, Nasveschuk C, Gagnon A, Chan EY, Sandy P, Busby J, Balasubramanian S, Campbell R, Zhao F, Bergeron L, Audia JE, Albrecht BK, Harmange JC (2013). Identification of EZH2 and EZH1 small molecule inhibitors with selective impact on diffuse large B cell lymphoma cell growth. Chem Biol.

[12] Knutson SK, Wigle TJ, Warholic NM, Sneeringer CJ, Allain CJ, Klaus CR, Sacks JD, Raimondi A, Majer CR, Song J, Scott MP, Jin L, Smith JJ (2012). A selective inhibitor of EZH2 blocks H3K27 methylation and kills mutant lymphoma cells. Nat Chem Biol.

[13] McCabe MT, Ott HM, Ganji G, Korenchuk S, Thompson C, Van Aller GS, Liu Y, Graves AP, Della Pietra A, Diaz E, LaFrance LV, Mellinger M, Duquenne C (2012). EZH2 inhibition as a therapeutic strategy for lymphoma with EZH2-activating mutations. Nature.

[14] Antonysamy S, Condon B, Druzina Z, Bonanno JB, Gheyi T, Zhang F, MacEwan I, Zhang A, Ashok S, Rodgers L, Russell M, Gately Luz J (2013). Structural context of disease-associated mutations and putative mechanism of autoinhibition revealed by X-ray crystallographic analysis of the EZH2-SET domain. PLoS One.

[15] Wu H, Zeng H, Dong A, Li F, He H, Senisterra G, Seitova A, Duan S, Brown PJ, Vedadi M, Arrowsmith CH, Schapira M (2013). Structure of the catalytic domain of EZH2 reveals conformational plasticity in cofactor and substrate binding sites and explains oncogenic mutations. PLoS One.

[16] Knutson SK, Kawano S, Minoshima Y, Warholic NM, Huang KC, Xiao Y, Kadowaki T, Uesugi M, Kuznetsov G, Kumar N, Wigle TJ, Klaus CR, Allain CJ (2014). Selective inhibition of EZH2 by EPZ-6438 leads to potent antitumor activity in EZH2-mutant non-Hodgkin lymphoma. Mol Cancer Ther.

[17] Epizyme (2015).

[18] O'Hare T, Eide CA, Deininger MW (2008). New Bcr-Abl inhibitors in chronic myeloid leukemia: keeping resistance in check. Expert Opin Investig Drugs.

[19] Lackner MR, Wilson TR, Settleman J (2012). Mechanisms of acquired resistance to targeted cancer therapies. Future Oncol.

[20] Garraway LA, Janne PA (2012). Circumventing cancer drug resistance in the era of personalized medicine. Cancer Discov.

[21] O'Hare T, Zabriskie MS, Eiring AM, Deininger MW (2012). Pushing the limits of targeted therapy in chronic myeloid leukaemia. Nat Rev Cancer.

[22] Gainor JF, Shaw AT (2013). Emerging paradigms in the development of resistance to tyrosine kinase inhibitors in lung cancer. J Clin Oncol.

[23] Arteaga CL, Engelman JA (2014). ERBB receptors: from oncogene discovery to basic science to mechanism-based cancer therapeutics. Cancer Cell.

[24] Kim E, Kim M, Woo DH, Shin Y, Shin J, Chang N, Oh YT, Kim H, Rheey J, Nakano I, Lee C, Joo KM, Rich JN (2013). Phosphorylation of EZH2 activates STAT3 signaling via STAT3 methylation and promotes tumorigenicity of glioblastoma stem-like cells. Cancer Cell.

[25] Vire E, Brenner C, Deplus R, Blanchon L, Fraga M, Didelot C, Morey L, Van Eynde A, Bernard D, Vanderwinden JM, Bollen M, Esteller M, Di Croce L (2006). The Polycomb group protein EZH2 directly controls DNA methylation. Nature.

[26] Bradley WD, Arora S, Busby J, Balasubramanian S, Gehling VS, Nasveschuk CG, Vaswani RG, Yuan CC, Hatton C, Zhao F, Williamson KE, Iyer P, Mendez J (2014). EZH2 inhibitor efficacy in non-Hodgkin's lymphoma does not require suppression of H3K27 monomethylation. Chem Biol.

[27] Gibaja V, Shen F, Harari J, Korn J, Ruddy D, Saenz-Vash V, Zhai H, Rejtar T, Paris CG, Yu Z, Lira M, King D, Qi W (2015). Development of secondary mutations in wild-type and mutant EZH2 alleles cooperates to confer resistance to EZH2 inhibitors. Oncogene.

[28] Bradeen HA, Eide CA, O'Hare T, Johnson KJ, Willis SG, Lee FY, Druker BJ, Deininger MW (2006). Comparison of imatinib mesylate, dasatinib (BMS-354825), and nilotinib (AMN107) in an N-ethyl-N-nitrosourea (ENU)-based mutagenesis screen: high efficacy of drug combinations. Blood.

[29] Zhou W, Ercan D, Chen L, Yun CH, Li D, Capelletti M, Cortot AB, Chirieac L, Iacob RE, Padera R, Engen JR, Wong KK, Eck MJ (2009). Novel mutant-selective EGFR kinase inhibitors against EGFR T790M. Nature.

[30] Zhang S, Wang F, Keats J, Zhu X, Ning Y, Wardwell SD, Moran L, Mohemmad QK, Anjum R, Wang Y, Narasimhan NI, Dalgarno D, Shakespeare WC (2011). Crizotinib-resistant mutants of EML4-ALK identified through an accelerated mutagenesis screen. Chem Biol Drug Des.

[31] Kim W, Bird GH, Neff T, Guo G, Kerenyi MA, Walensky LD, Orkin SH (2013). Targeted disruption of the EZH2-EED complex inhibits EZH2-dependent cancer. Nat Chem Biol.

