# Supplementary material for: Acquisition of a single EZH2 D1 domain mutation confers acquired resistance to EZH2-targeted inhibitors
Source: Oncotarget. 2015 Sep 2;6(32):32646–55. doi: 10.18632/oncotarget.5066 (PMC4741719; doi:10.18632/oncotarget.5066)
Supplement: Supplementary file 1 [file oncotarget-06-32646-s001.pdf]

SUPPLEMENTARY FIGURES

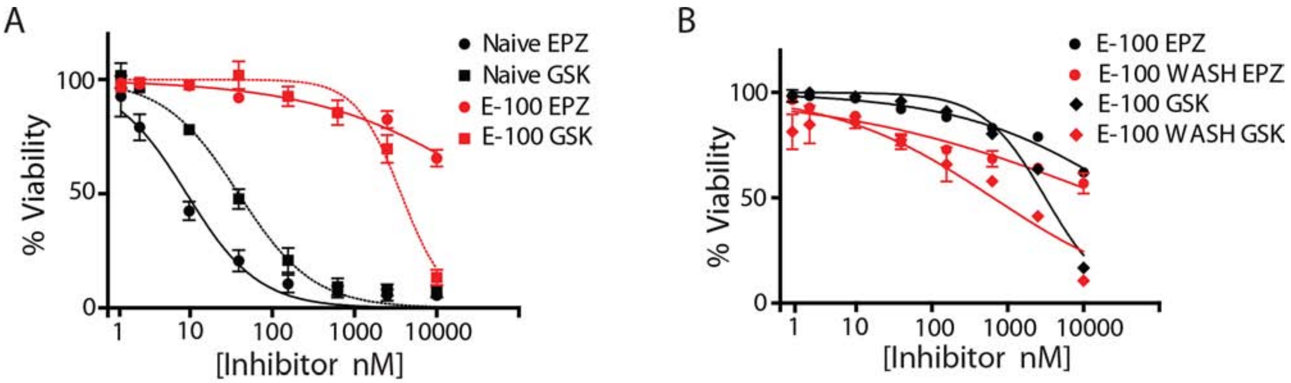

C

		E-100
Unwash	EPZ-6438	>10000
	GSK126	4030±370
Wash 3 weeks	EPZ-6438	>10000
	GSK126	1222±89

IC50 (nM)

Supplementary Figure S1: Characterization of B cell lymphoma cell lines sensitive and resistant to EZH2i. A. Sensitivity of E-100 Pfeiffer cells to a dose response of EPZ-6438 (EPZ) and GSK 126 (GSK). **B.** E-100 cells grown in absence (WASH) of EZH2i for 8 weeks were treated with a dose response of EZH2i and assayed for cell viability. **C.** All IC_{50} values (±S.D.) are calculated from three independent viability assays.

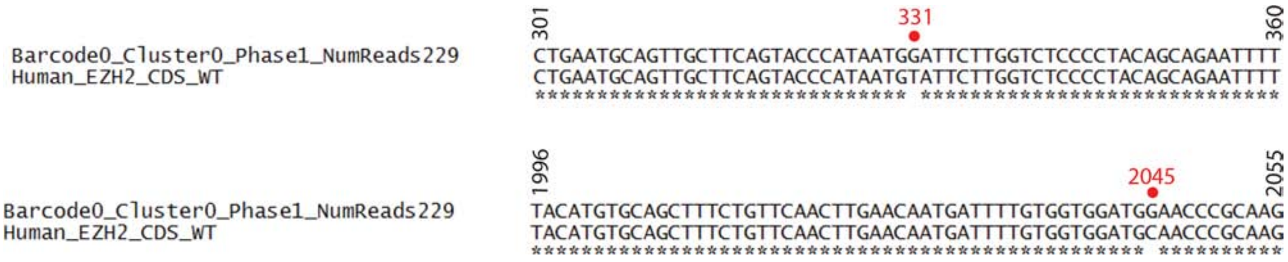

Supplementary Figure S2: Y111D and A677G mutations are located within the same allele. Alignment of the phase 1 single molecule sequencing read around the Y111D and A677G region with EZH2 WT CDS. No mutations were identified in the phase 0 sequencing reads (data not shown). Missense mutations are highlighted in red.

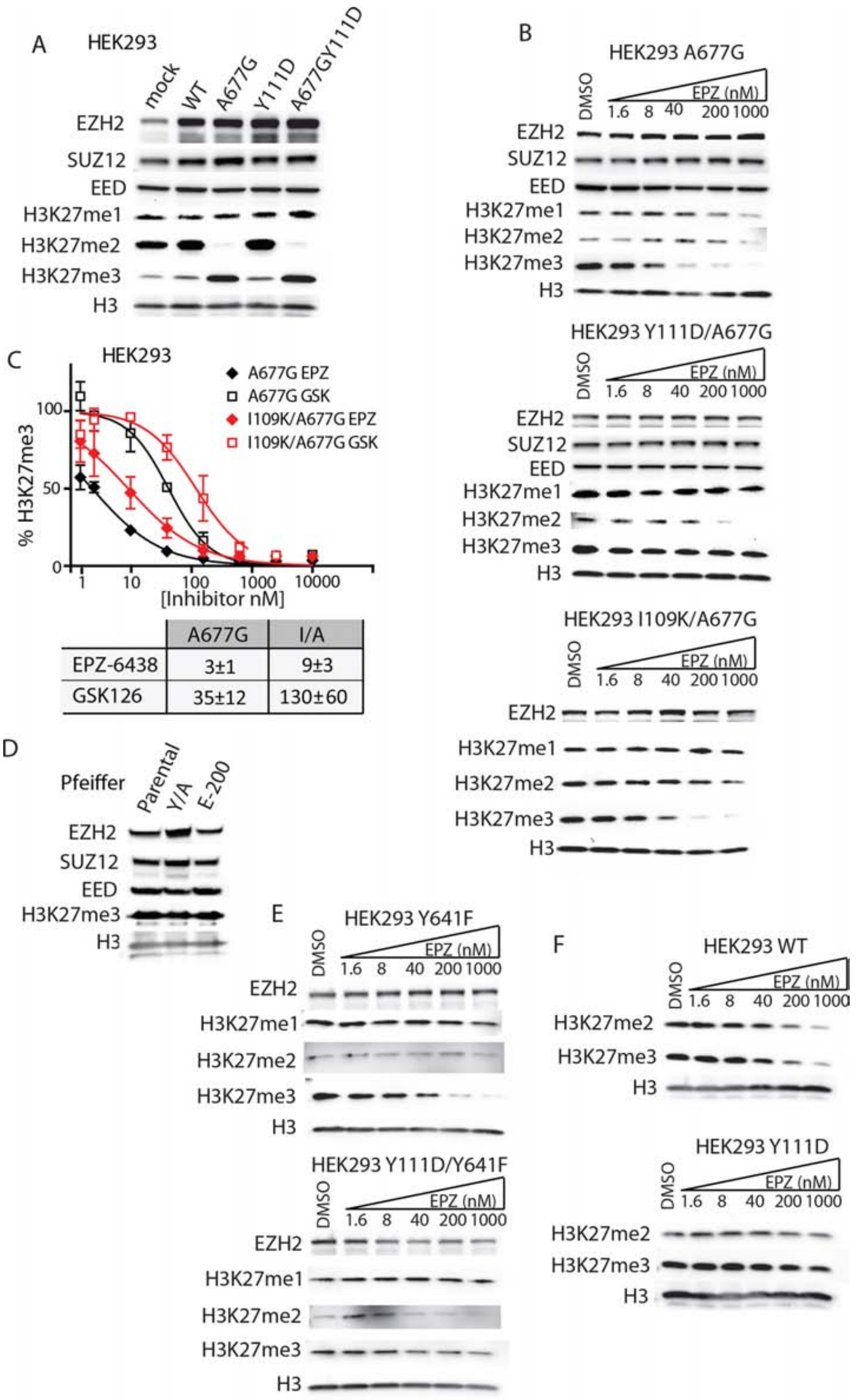

Supplementary Figure S3: A single EZH2 D1 domain mutation confers resistance to EZH2i. A. HEK293 cells were transfected with WT or indicated EZH2 mutants and analyzed by immunoblot for EZH2, EED, SUZ12 and H3K27 methylation status. **B, E,** and **F.** HEK293 cells stably expressing A677G (**B**), Y111D/A677G (**B**), I109K/A677G (**B**), Y641F (**E**), Y111D/Y641F (**E**), WT EZH2 (**F**) and Y111D (**F**) mutants were treated with a dose response of EPZ-6438 and analyzed by immunoblot for EZH2, EED, SUZ12 and H3K27 methylation status. Total histone H3 was used as a loading control. **C.** Measure of H3K27me3 in EZH2 mutants-expressing HEK293 following treatment with EPZ-6438 (EPZ) and GSK 126 (GSK). IC_{50} values (±S.D.) were calculated from three independent H3K27me3 alpha-LISA experiments. **D.** Parental, E-200, and Y111D/A677G stably expressing Pfeiffer cells were analyzed by immunoblot for EZH2, EED, SUZ12, and H3K27me3 levels.

A

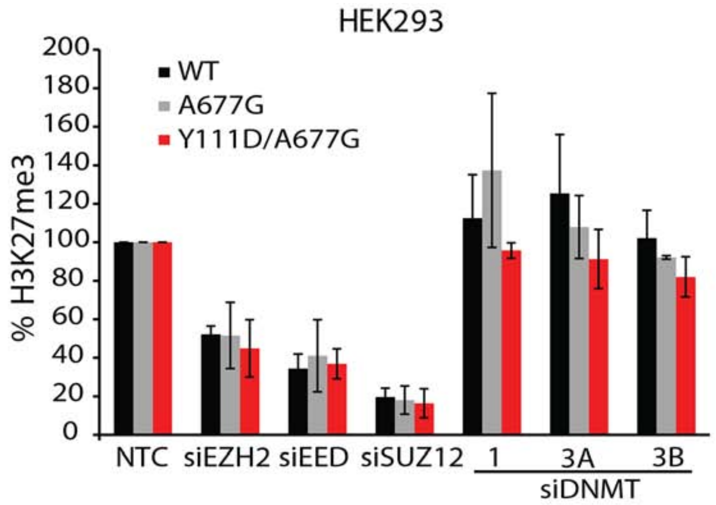

B

Pfeiffer	parental	EZH2 constructs: WT	Y111D	Y/A	Y641	Y/Y	Y/A/H
EPZ-6438	7±1	6±3	6±3	>10000	7±3	28±8	5±1
GSK126	23±5	31±18	39±12	2673±1373	26±12	111±35	25±9

IC50 (nM)

Supplementary Figure S4: Y111D activity is dependent on PRC2 complex components. A. HEK293 cells stably expressing WT or indicated EZH2 mutants were transfected with siRNA against the PRC2 subunits (EZH2, EED, and SUZ12) or DNMTs (1, 3A, or 3B) and assayed for H3K27me3 levels. **B.** Sensitivity of Pfeiffer stably-expressing WT and mutants EZH2. IC_{50} values (±S.D.) are calculated from three independent viability assays.
